# Polyvinylpyrrolidone‐Coordinated Single‐Site Platinum Catalyst Exhibits High Activity for Hydrogen Evolution Reaction

**DOI:** 10.1002/anie.202005282

**Published:** 2020-06-29

**Authors:** Can Li, Zheng Chen, Hong Yi, Yi Cao, Lei Du, Yidong Hu, Fanpeng Kong, Richard Kramer Campen, Yunzhi Gao, Chunyu Du, Geping Yin, Igor Ying Zhang, Yujin Tong

**Affiliations:** ^1^ MIIT Key Laboratory of Critical Materials Technology for New Energy Conversion and Storage Harbin Institute of Technology Harbin 150001 China; ^2^ Fritz Haber Institute of the Max Planck Society Faradayweg 4–6 14195 Berlin Germany; ^3^ Shanghai Key Laboratory of Molecular Catalysis and Innovative Materials Collaborative Innovation Center of Chemistry for Energy Materials MOE Laboratory for Computational Physical Science Department of Chemistry Fudan University 200433 Shanghai China; ^4^ College of Chemistry and Molecular Sciences Wuhan University Wuhan 430072 China; ^5^ Department of Chemistry Hefei National Laboratory for Physical Sciences at the Microscale iChEM (Collaborative Innovation Center of Chemistry for Energy Materials) University of Science and Technology of China Hefei 230026 China; ^6^ Faculty of Physics University of Duisburg-Essen Lotharstraße 1 47057 Duisburg Germany

**Keywords:** electrocatalysis, electrochemistry, hydrogen evolution reaction, photochemistry, water splitting

## Abstract

The essence of developing a Pt‐based single‐atom catalyst (SAC) for hydrogen evolution reaction (HER) is the preparation of well‐defined and stable single Pt sites with desired electrocatalytic efficacy. Herein, we report a facile approach to generate uniformly dispersed Pt sites with outstanding HER performance via a photochemical reduction method using polyvinylpyrrolidone (PVP) molecules as the key additive to significantly simplify the synthesis and enhance the catalytic performance. The as‐prepared catalyst displays remarkable kinetic activities (20 times higher current density than the commercially available Pt/C) with excellent stability (76.3 % of its initial activity after 5000 cycles) for HER. EXAFS measurements and DFT calculations demonstrate a synergetic effect, where the PVP ligands and the support together modulate the electronic structure of the Pt atoms, which optimize the hydrogen adsorption energy, resulting in a considerably improved HER activity.

## Graphical abstract

**Ligand‐like**, polyvinylpyrrolidone molecules stabilize single‐atom Pt during UV‐induced photoreduction on titanium oxide nanorods encapsulated by graphitic carbon. The resulting material shows extremely high activity toward the hydrogen evolution reaction.

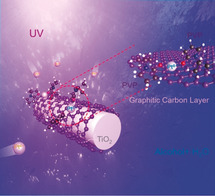

Single‐atom catalysts (SACs) have attracted increasing and extensive attention in heterogeneous catalysis, owing to their ultimate high atom utilization efficiency and unparalleled excellent catalytic performance.^1^, ^2^, ^3^, ^4^ Especially for noble metal based SACs, the outstanding catalytic activity may outweigh their scarcity and high cost, thereby allow large‐scale application.^5^

Pt‐based electrocatalysts are the most active catalysts for the electrochemical hydrogen evolution reaction (HER), which is one of the most important and extensively studied electrochemical half‐reactions for the hydrogen economy. To date, atomically isolated Pt atoms have been successfully anchored on various support materials and exhibited enhanced catalytic performances in comparison to Pt nanoparticles.^6^, ^7^, ^8^, ^9^, ^10^, ^11^, ^12^, ^13^ However, the majority of the reported approaches for preparing SACs require high‐temperature pyrolysis,^6^, ^7^, ^8^, ^9^ inevitably leading to mutational structural changes, randomness, and often aggregation.^14^ In addition, the interaction of the metal atoms with the environment, which has been considered as a vital parameter for tuning the single‐site catalytic activity, is still largely unknown and usually not well defined, particularly for the SACs prepared via pyrolysis methods. It is crucial, yet remains a great challenge, to develop a practical approach under mild reaction conditions with a well‐controlled atom–substrate interaction scheme.

Photochemical reduction has proven to be an effective alternative for the preparation of high‐quality SACs.^15^, ^16^ In order to prevent the migration and aggregation of the active Pt atoms, most of the photochemical methods require freezing the precursor solution under harsh preparation conditions,^16^, ^17^ which, however, is impractical for industrial applications. Furthermore, the reduction mechanisms for many of the reported photochemical reactions are unclear: Since either a broadband UV/Vis lamp^17^ or a UV lamp with photon energy out of the absorption band of the reactant^18^ was employed, the underlying reduction mechanisms are intricate and hard to understand. In this study, we introduce titanium oxide nanorods encapsulated by graphitic carbon (TNR@GC) to a precursor solution containing hexachloroplatinate and irradiated it with a 365 nm UV monochromatic lamp. In this way, the positively charged Pt atoms in the solution can be indirectly reduced by the photoexcited electron of the TNR. To prevent the aggregation of the Pt atoms during reduction process without employing the freezing strategy and to further manipulate the electronic structure of the Pt atoms with a desired coordination structure,^19^, ^20^, ^21^, ^22^ we added the organic ligand polyvinylpyrrolidone (PVP) to the photochemical system. PVP has been well studied as an organic ligand to both stabilize noble metal nanoparticles and regulate the interfacial electronic structure.^22^, ^23^, ^24^ While PVP is unstable under UV irradiation with high‐energy photons (185 and 257 nm),^25^, ^26^, ^27^ the 365 nm UV light used in the present study is far from the center energy of the UV absorption peak of PVP^28^, ^29^ and hence does not cause decomposition. We found that the PVP molecules can attach to the TNR@GC substrate through van der Waals interactions (Scheme 1). The pyrrolidone side chain tends to interact with the photoreduced Pt atoms under UV light irradiation to form a stable complex. The complex together with the substrate form a novel catalyst which exhibits superior HER electrocatalytic performance. With the help of the density functional theory (DFT) simulations and atomic level structural characterization tools, such as high‐resolution transmission electron microscopy (HRTEM), X‐ray absorption near‐edge structure (XANES), and extended X‐ray absorption fine structure (EXAFS), we were able to understand the reaction mechanism and reveal how the local electronic structure of the single Pt metal site is tuned by the coordination environment and how the system as a whole leads to improved HER reactivity. The concept of employing organic ligands to stabilize the photoreduction product in this study can provides a new avenue for rationally designing highly stable and active single‐atom catalysts for various catalytic reactions and large‐scale industrial applications.

**Scheme 1:**
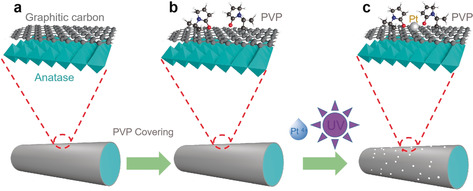
Illustration of the synthesis of Pt‐PVP/TNR@GC catalysts.

## Results and Discussion

The procedures to synthesize the PVP (K30)‐coordinated Pt single‐site TNR@GC catalysts are summarized in Scheme 1; a detailed description of the reaction conditions and the properties of the catalyst support are given in the Supporting Information (Figures S1–S3) In brief, a narrow band of 365 nm ultraviolet light was introduced to indirectly reduce the Pt^4+^ cations via the photoexcited electron of the TiO_2_ substrate. FTIR measurements were conducted before and after UV irradiation to confirm the stability of the PVP.

**Figure 1:**
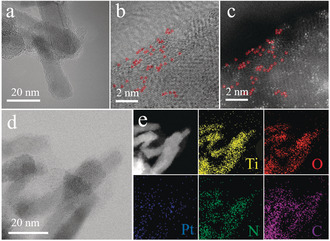
a) HRTEM image of TNR@GC, b) ABF‐STEM image of Pt‐PVP/TNR@GC, and c) AC HAADF‐STEM image of the same region (Pt atom positions are indicated by red circles), d) TEM image of Pt‐PVP/TNR@GC, and e) corresponding element mapping.

To verify the atomic Pt site distribution on the Pt‐PVP/TNR@GC complex, we performed aberration‐corrected high‐angle annular bright/dark field‐scanning transmission electron microscopy (ABF‐/HAADF‐STEM). Figure 1 b,c clearly show the isolated Pt atoms on Pt‐PVP/TNR@GC as indicated by the bright spots highlighted in red. Large‐scale TEM images and XRD patterns also reveal no obvious nanoparticles or clusters in Pt‐PVP/TNR@GC (Figure 1 b,c and Figure S4). The HAADF‐STEM image as well as element mapping in Figure 1 e (same region as in Figure 1 d) clearly demonstrate that the Pt, N, C, Ti, and O elements have been distributed homogeneously over the catalyst. The Pt loading was measured to be 1.75 wt % by inductively coupled plasma optical emission spectroscopy (ICP‐OES). This value is much higher than the Pt loading of Pt SACs prepared by the simple wetness impregnation method (<1 % wt %)^30^, ^31^, ^32^, ^33^ and comparable to that of catalysts prepared by the thermal emitting method (≈2.1 % wt %).^7^

**Figure 2:**
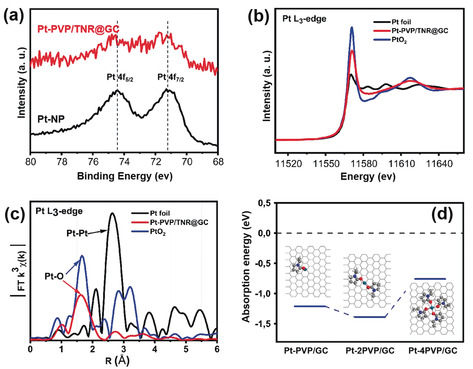
a) XPS spectrum of Pt‐PVP/TNR@GC and Pt‐NP b) Pt K‐edge XANES spectra and c) R‐space EXAFS spectra of Pt foil, PtO_2_, and Pt‐PVP/TNR@GC. d) Absorption energies of various kinds of PVP‐coordinated Pt single sites on the graphitic carbon surface.

The valence states of the isolated Pt atoms on Pt‐PVP/TNR@GC were investigated by X‐ray photoelectron spectroscopy (XPS). As shown in Figure 2 a, the binding energies of the Pt 4f spectra for Pt‐PVP/TNR@GC are 72.3 eV and 75.8 eV (denoted as Pt^δ+^), which can be assigned to Pt 4f_7/2_ and Pt 4f_5/2_, respectively. The Pt^δ+^ peaks are located between those of Pt^0^ and Pt^4+^ (0<*δ*<4), indicating that the isolated Pt atoms in Pt‐PVP/TNR@GC possess a more positive valence state than those in Pt nanoparticles. Such a partial charged state can be attributed to the strong interaction between Pt and the pyrrolidone group of the PVP molecules in the form of Pt–O ligand bonds (see the discussion below for more details).

To further identify the coordination environment and the electronic structure of the single Pt atoms, we conducted XANES and EXAFS measurements. The XANES spectra of Pt‐PVP/TNR@GC, Pt foil, and PtO_2_ are shown in Figure 2 b. (Fitting of the main peak and the corresponding parameters can be found in Figure S5 and Table S2 of the Supporting Information.) The white line intensity of Pt‐PVP/TNR@GC is much higher than that of Pt foil and is evidently lower than that of PtO_2_, confirming the oxidation state of Pt^δ+^ (0<δ<4). The Pt L_3_‐edge EXAFS Fourier transform (FT) spectrum of Pt‐PVP/TNR@GC (Figure 2 c) exhibits a prominent peak around 1.65 Å, which is similar to that of the Pt−O bond in PtO_2_ and is about 1 Å shorter than that of Pt–Pt (2.64 Å) in Pt foil. The DFT calculations indicate that the single Pt sites coordinated by two pyrrolidone groups are the most stable structures on the graphitic carbon surface (Figure 2 d, see the Supporting Information for details of the simulations).

**Figure 3:**
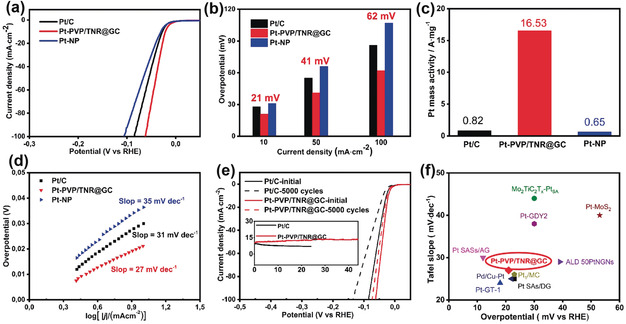
a) HER linear sweep voltammetry (LSV) curves of various catalysts in 0.5 m H_2_SO_4_, scan rate 5 mV s^−1^. b) Overpotential of catalyst at 10, 50, and 100 mA cm^−2^. c) Mass activities at −0.05 V of catalysts for the HER in 0.5 m H_2_SO_4_. d) Corresponding Tafel plots. e) Stability test of Pt‐PVP/TNR@GC in 0.5 m H_2_SO_4_ through potential cycling, before and after 5000 cycles; inset: chronoamperometry curves of Pt‐PVP/TNR@GC at −0.021 V for 44 h and Pt/C at −0.028 V for 24 h. f) Comparison with other reported electrocatalysts in 0.5 m H_2_SO_4_.

The electrocatalytic activity of Pt‐PVP/TNR@GC for HER was evaluated in H_2_‐saturated 0.5 m H_2_SO_4_ using a standard three‐electrode setup (a graphite rod served as the counter electrode). Pt‐NP and commercial Pt/C were also measured under the same conditions for comparison. As shown in Figure 3 a, the HER linear sweep voltammetry (LSV) curves of Pt‐PVP/TNR@GC exhibit a substantially lower hydrogen overpotential (21 mV) than Pt‐NP (31 mV) and Pt/C (28 mV), at a current density of 10 mA cm^−2^. All the potentials have been iR‐corrected according to the electrochemical impedance spectroscopy (EIS) curves (see Figure S6 in the Supporting Information). Notably, the mass current density for Pt‐PVP/TNR@GC is 16.53 A mg^−1^, 25 and 20 times higher than that of Pt‐NP (0.65 A mg^−1^) and Pt/C (0.82 A mg^−1^) (measured at −0.05 V vs. reversible hydrogen electrode (RHE)) respectively. Meanwhile, Pt‐PVP/TNR@GC achieves the corresponding Tafel slope of 27 mV dec^−1^, demonstrating more favorable kinetics than those of Pt‐NP (34 mV dec^−1^) and commercial Pt/C (31 mV dec^−1^). Furthermore, Pt‐PVP/TNR@GC also exhibits a higher HER activity than Pt/C in alkaline media (Figure S7).

In addition, the catalytic performance of Pt‐PVP/TNR@GC shows no significant decay after 5000 CV cycles (Figure 3 e and Figure S8). Next, the chronoamperometry curve of Pt‐PVP/TNR@GC shows a negligible attenuation at −21 mV after 44 hours (Figure 3 e inset). HAADF‐STEM images, and EXAFS and XRD spectra of Pt‐PVP/TNR@GC confirmed the atomically isolated, dispersed Pt single sites retained after the HER stability tests (Figure S9, S10 and S4c). The above results indicate that Pt‐PVP/TNR@GC possesses an excellent HER catalytic stability.

**Figure 4:**
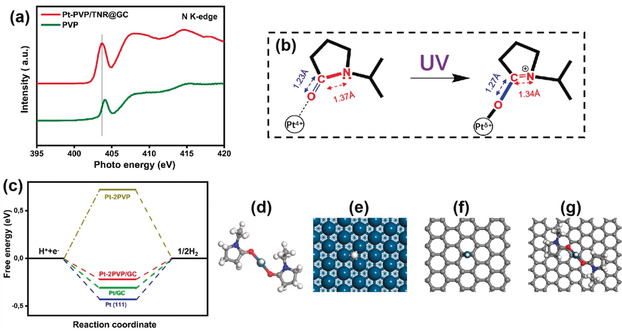
a) N K‐edge XANES spectra of PVP and Pt‐PVP/TNR@GC. b) Change in the local structure of a Pt atom coordinated by PVP before and after UV irradiation; bond lengths were obtained from DFT calculations, see Table S3 in the Supporting Information for details. c) Free energy diagram of hydrogen evolution at zero potential and pH 0 for Pt‐2PVP/GC, Pt‐2PVP, Pt/GC, and Pt(111). d–g) The optimized structures of PVP coordinated to Pt atoms in the gas phase (Pt‐2PVP) and in Pt(111), (Pt/GC), and Pt‐2PVP supported on graphitic carbon (Pt‐2PVP/GC) with adsorbed H, respectively. White, gray, blue, red, and cyan balls represent H, C, N, O, and Pt atoms, respectively. Graphitic carbon is modeled by graphene in the DFT calculations.

Synchrotron‐based XANES analysis of the K‐edge of the N element was employed to further study the local coordination structure of the single Pt sites. Figure 4 a shows that the normalized N K‐edge XANES spectra of Pt‐PVP/TNR@GC and the reference bare PVP exhibit similar spectral features. The spectrum can be divided into two separate regions: the sharp peak located at ≈404 eV and two broad features around 408 eV and 415 eV. The former can be assigned to the N 1s→π* transition^34^, ^35^, ^36^ and the latter two to N 1s→σ* N‐C transitions.^37^, ^38^ Comparing with the spectrum of bare PVP, the redshift of the N 1s→π* transition and the enhanced intensity of the N 1s→σ* N‐C transitions of Pt‐PVP/TNR@GC can be understood as a result of the N−C bond in Pt‐PVP/TNR@GC being more significantly integrated into the strongly electron‐withdrawing (C=O‐Pt) group when coordinated with Pt atoms.^39^, ^40^ This result is in good accordance with the observation from our DFT simulations that: 1) the calculated partial density of states (PDOSs) of the N atoms give clear evidence of the disappearance of the lone pair electrons of the N atoms in the pyrrolidone group after coordination with Pt atoms to form the Pt‐PVP complex (Supporting Information Figure S11); and 2) the calculated N−C bond length in the Pt‐coordinated PVP decreases from 1.37 Å to 1.34 Å in company with the increase of the C=O bond length from 1.23 Å to 1.27 Å. (Figure 4 b).

We used the model of the Pt‐PVP complex on an ideal graphitic carbon surface (Pt‐PVP/GC) in our DFT calculations, which suggest that the twofold PVP‐coordinated Pt single atom is the most stable complex on the support; it is −1.36 eV more stable than isolated PVP and the bare Pt atom on graphene (Figure 4 c). This finding is consistent with the experimental observations that without the PVP coordination, Pt atoms tend to form nanoparticles during the photochemical reduction.^41^ The Gibbs free energies for hydrogen adsorption Δ*G*_(H*)_ were calculated over the three models as shown in Figure 4 c.^42^, ^43^ Interestingly, Pt‐2PVP/GC presents the lowest |Δ*G*_(H*)_| in comparison to the Pt/GC and Pt‐2PVP models, which yield a stronger hydrogen adsorption and a repulsive interaction, respectively. This observation not only confirms the enhanced HER activity of Pt‐PVP/TNR@GC obtained in experiment, but also implies that the synergy effect between PVP ligands and the GC surface is the key to optimize the catalytic performance of Pt‐PVP/TNR@GC. Along this line, we argue that the catalytic performance of Pt‐based SACs can be further improved by searching for other organic ligands that can deliver a stronger repulsive contribution to the hydrogen adsorption energy.

## Conclusion

In conclusion, a well‐controlled photochemical process has been applied to produce a Pt SAC in a pyrolysis‐free manner. The novel strategy allows us to optimize the electronic structure of the single‐atom platinum sites via organic stabilizers. The abundant amide functional groups of PVP not only serve as the coordination environment to fix the Pt atoms, but also modulate the electronic structure. Both theoretical and experimental studies have shown that a partially positively charged Pt atom coordinated by a ‐C=O moiety of the pyrrolidone side chain is the active center for the HER. Pt SACs prepared in this study show impressive performance and high stability for the hydrogen evolution reaction. This approach offers general and valuable insights for the design of noble‐metal‐based single‐atom catalysts (such as Au, Pd, Ru, Ag, etc.) and optimization of catalysis performance toward various chemical reactions, provided the ligand groups are carefully chosen based on the electronic structure of both the noble metal and the ligand.

## Conflict of interest

The authors declare no conflict of interest.

## Supporting information

As a service to our authors and readers, this journal provides supporting information supplied by the authors. Such materials are peer reviewed and may be re‐organized for online delivery, but are not copy‐edited or typeset. Technical support issues arising from supporting information (other than missing files) should be addressed to the authors.

Supplementary
