# Supplementary material for: Polyvinylpyrrolidone‐Coordinated Single‐Site Platinum Catalyst Exhibits High Activity for Hydrogen Evolution Reaction
Source: Angew Chem Int Ed Engl. 2020 Jun 29;59(37):15902–7. doi: 10.1002/anie.202005282 (PMC7539980; doi:10.1002/anie.202005282)
Supplement: Supplementary file 1 — Supplementary [file ANIE-59-15902-s001.pdf]

Angewandte Chemie
Eine Zeitschrift der Gesellschaft Deutscher Chemiker

Supporting Information

Polyvinylpyrrolidone-Coordinated Single-Site Platinum Catalyst Exhibits High Activity for Hydrogen Evolution Reaction

Can Li[+], Zheng Chen[+], Hong Yi, Yi Cao, Lei Du, Yidong Hu, Fanpeng Kong, Richard Kramer Campen, Yunzhi Gao, Chunyu Du, Geping Yin,* Igor Ying Zhang,* and Yujin Tong**

anie_202005282_sm_miscellaneous_information.pdf

This PDF file includes the follow content:

Materials and Methods

Supplementary Figures S1-S11

Tables S1-S3

References

Materials and Methods

Chemicals

All the chemicals were of analytical grade and used as received without further purification. Titanium dioxide (TiO_2, P25), D-Glucose ($C_6H_{12}O_6$), sodium sulfate (Na_2SO_4), and poly(N-vinylpyrrolidone) (PVP, K30), Mw~58 000, were purchased from Aladdin Industrial Corporation; Chloroplatinic acid hexahydrate ($H_2PtCl_6 \cdot 6H_2O$), were purchased from Sigma-Aldrich; Sodium hydroxide (NaOH), hydrochloric Acid (HCl ~35wt%), sulfuric acid (H_2SO_4 ~98wt%), Ethyl alcohol were purchased from Sinopharm Chemical Reagent Co. Ltd. (Shanghai, China); Nafion solution (5.0wt%) was purchased from Dupont; and the benchmark Pt/C (20wt%) was purchased from Alfa Aesar; Deionized water (18.2 MΩ, Mill-Q Corp.) was used in the whole experiment.

Synthesis of TNR@GC.

The graphitic carbon encapsulated titanium dioxide nanorods (TNR@GC) support has been reported in our previous study,[30] High-resolution transmission electron microscopy (HRTEM) image (Figure 1a) shows that TNR@GC has rod-like structure with an ultra-thin carbon encapsulation layer. XRD data (Figure S1) indicates that the TNR@GC exhibits anatase TiO_2 characteristic peaks (Scheme 1a). In a typical synthesis, 100 mg TiO_2 (P25) powers were added into NaOH aqueous solution (80 ml, 10 M) under magnetic stirring at room temperature

for 2 h. Then the mixture was added to a 100 ml Teflon-lined autoclave and kept at 130 °C for 24 h. The precipitates were washed by distilled water several times until the pH of the solution reached 7 and dried in a vacuum at 80 °C for overnight to obtain the titanium dioxide nanorods (donated as TNR).

A certain mass ratio (Ti/C=1:3) of glucose and TNR were dissolved in the water to form a suspension, then the mixed solution transferred to a Teflon-lined autoclave followed by the second hydrothermal treatment at 180 °C for 12 h under constant rotating conditions. After the hydrothermal reaction, the products were filtered and dried at 80 °C for 5 h. Finally, the samples were calcined at 800 °C for 2 h (in 10% H_2/ Ar) to obtain the graphite-like graphitic carbon encapsulated titanium dioxide nanorods (donated as TNR@GC).

Synthesis of Pt-NP

Pt nanoparticles deposited on the graphite carbon encapsulated TNR (Pt-NP) were synthesized in aqueous solution via UV irradiation at room temperature with an Ar atmosphere. Typically, 40 mg of TNR@GC was dispersed into mixed solution (40 mL) containing ethyl alcohol and deionized water at a volume ratio of 1:1, the mixture was sonicated for 30 minutes and then magnetically stirred for one hour. Then an appropriate amount of platinum precursor was added under vigorous stirring. After being saturated with argon, the photo-reduction takes place under a high-pressure Hg lamps with 365 nm UV irradiation for 30 minutes. Finally, the products were filtered and washed with deionized water several times, then dried overnight in a vacuum oven at 80 °C.

Synthesis of Pt-PVP/TNR@GC

The above as-prepared TNR@GC powder was used as the support for the subsequent Pt SACs preparation. To avoid the migration and agglomeration of photo-reduced Pt atoms on the support and to achieve a high loading of Pt single sites, here we chose the polyvinylpyrrolidone (PVP, K30) molecules to cover the TNR@GC surface by mixing 15 mg PVP and 20 mg TNR@GC powder in a 40 ml solution containing H_2O (50 %) and ethanol (50 %) with 12 hour

stirring (Scheme 1b). Subsequently, $H_2PtCl_6{\cdot}6H_2O$ solution (75uL, 38.97mM) was injected into the above mixture and stirred for 8 h in the dark. It needs to point out that the amount of PVP on the catalysts is determined by the amount of the TNR@GC support. As mentioned in the text, the interaction between the PVP and the TNR@GC support is via Van der Waals interaction. We found that the coverage of PVP is saturated when the PVP and the TNR@GC ratio is about 3:4 in weight. See Table S1 below, the excess PVP molecules were leached out by the washing processes with alcohol and DI water after centrifuging. During this process, the $PtCl_6^{2-}$ anions interact with the pyrrolidone side chains of PVP resulting in an atomic isolation of Pt atoms.[24] To reduce the Pt^{4+} cations, we adopted the similar photochemical reduction strategy as reported previously,[30] i.e. irradiating the sample with a narrow band 365 nm ultraviolet light (full width of the half maximum: FWHM ~2nm) for 30 minutes at 30°C in an argon atmosphere. The photon energy of the UV (365 nm, 3.40 ev) was selected to avoid both the decomposition of PVP by the UV-ozone[25-27] and the direct reduction of the Pt^{4+} cations from solution but was sufficient to drive electron/hole separation in the TiO_2 substrate (the spectrum of the UV light can be found in Figure S2). The photo excited electrons reduce the Pt4+ cations surrounding the TNR@GC while the holes are consumed by the ethanol. The reaction took place under the UV irradiation (~4.8 $mW{\cdot}cm^{-2}$) at 30 °C in a thermostat for 30 minutes. The resultant slurry was centrifuged and washed in alcohol and DI water four times. Finally, the atomically dispersed Pt was obtained by dried overnight in a vacuum oven at 80 °C. To confirm that the 365 nm UV light used in our study does not cause decomposition of the PVP, we have also conducted FTIR measurements before and after UV irradiation (Figure. S3 in the supporting information). Afterward, the solution was centrifuged and washed with alcohol and subsequently de-ionized water. The single atom Pt catalysts were thus obtained (Scheme 1c).

Material Characterization:

X-ray diffraction patterns was performed on a Rigaku D/max-γA X-ray diffractometer at 4° min^{-1} scanning speed. The operating voltage and current were 40 kV and 15 mA with Cuκα radiation (λ=1.54178 Å). Transmission electron microscopy (TEM) images and high resolution

TEM (HRTEM) images were recorded on a TecnaiG2F30 at 200 kV. Aberration-corrected High-angle annular dark-field scanning transmission electron microscopy (HAADF-STEM) images and corresponding EELS mapping were recorded by a JEOL JEMARM200F TEM/STEM system operated at a voltage of 200 kV. The composition of Pt was measured on ICP-OES measurements (iCAP 6300 Thermo). Fourier transform infrared spectra (FT-IR) of the samples were recorded on a VECTOR-22FT-IR spectrometer over potassium bromide pellet. X-ray photoelectron spectroscopy (XPS) was carried out by an Al Ka (hν =1486.6 eV) radiation source (Thermo ESCALAB 250). Electron paramagnetic resonance (EPR) spectra were performed by a Bruker ELEXSYS E500 EPR spectrometer at room temperature. The UV-vis absorption spectra was conducted on a SolidSpec 3700 (range: λ=200~800 nm, $BaSO_4$ as the reflectance standard).

The X-ray absorption fine structure spectra (XAFS) measurements at the Pt L_3-edge were performed in both transmission (Pt foil) and fluorescence (the samples) modes at beamline 1W1B station (Beijing Synchrotron Radiation Facility). The electron beam energy was 3.5 GeV, and the maximum stored current was 260 mA. The fluorescence signal of Pt L_3-edge was collected by a Lytle detector, and a Pt foil was used to calibrate the energy. A fixed-exit double-crystal Si (111) monochromator was used to collect XAFS data in ambient conditions. The obtained EXAFS data were conducted according to the standard procedures using the IFEFFIT software package. The EXAFS spectra were calibrated and averaged, the pre-edge background was subtracted, and the post-edge was normalized using the Athena module implemented in the IFEFFIT software package. The k^3-weighted $\chi(k)$ data of Pt L_3-edge was Fourier transformed to R space using a hanning windows (dk=1.0 $Å^{-1}$) to separate the EXAFS contributions from different coordination shells. The data fitting was performed using the Artemis program module of the IFEFFIT package. The Soft X-ray absorption spectra (N K-edge) was collected at BL 12B X-ray Magnetic Circular Dichroism (XMCD).

Electrochemical measurements

All electrochemical experiments were conducted in a three-electrode system using an

electrochemical workstation (CHI 760D) in 0.5 M H_2SO_4 at room temperature. Samples were measured on a rotating-disk glassy-carbon electrode (RDE, 5 mm diameter, 0.196 cm^2) and used as the working electrode, $Hg/HgSO_4$ electrode as the reference electrode and a graphite rod as the counter electrode.

The catalyst ink were prepared by mixing 5 mg catalysts with a mixture (490μL ethanol, 490μL H_2O and 40μL 5% Nafion solution (DuPont)) under ultrasonic conditions for 1 hour. Then, 50 uL of the well-dispersed catalyst ink was uniformly dropped onto freshly polished RDE with drying naturally for next test. The HER performance was conducted in H_2-saturated 0.5 M H_2SO_4 solution with a scan rate of 5 $mV \cdot s^{-1}$. All potential vs. Hg/HgSO4 was calibrated to the reversible hydrogen electrode (RHE) according to the equation (E_{RHE}=E_{test} -$E_{Ag/AgCl}$-I * Rs). Electrochemical impedance spectroscopy (EIS) tests were carried out from 100 kHz to 0.1 Hz with an amplitude of 5 mV at -0.021 V in H_2SO_4. The electrochemical durability stability was evaluated by 5, 000 cycle voltammetry sweeps (range of 0.2 V and −0.1 V) at 50 $mV \cdot s^{-1}$ in 0.5 M H_2SO_4 solution. Chronoamperometry measurement was conducted at the potential of −0.021 V for 44 hours. The working electrode kept rotating at 1,600 rpm to remove the H_2 generated at the electrode surface.

Computational details

The Vienna *ab initio* simulation package (VASP) was utilized to perform all density functional theory (DFT) calculations.[1-3] The 2s, 2p electrons in carbon, nitrogen and oxygen and the 5d, 6s electrons in platinum were treated as valence electrons, while the kinetic energy cutoff for the plane wave basis sets was set to be 400 eV. The remaining core electrons were described by the projector augmented-wave (PAW) method.[4] The surface Monkhorst–Pack meshes[5] of 2 × 2 × 1 k-point sampling in the surface Brillouin zone were employed for the slab model. For pristine Pt(111) surface, a 4 × 4 (11.09 Å × 11.09Å) supercell of 3 fixed bottom atomic layers and 3 relaxed top atomic layers was used. For the pristine graphene, the graphene basal plane model with a supercell 8 × 4√3 (19.70 Å × 17.07Å) containing 128 C atoms was used. After the convergence criterion for optimizations has been met, the largest remaining force on each

atom is less than 0.02 eV Å^{-1}. For all calculations, the generalized gradient approximation (GGA) of the Perdew–Burke–Ernzerhof (PBE) functional[6] was used. The contribution of dispersive interactions are accounted for by using the DFT+D3 method with Becke-Jonson (BJ) damping.[7-8] Bader's theory of atoms in molecules is used for charge analysis.[9-10]

Here we use monomer N-vinylpyrrolidone instead of polymer PVP to build our unit cell, the justification is given below: this is in part because the electronic correlation effect is often nearsightedness[11-12] and the (electro-)catalysis performance of transition-metal complexes is mainly driven by the ligand bonding interactions in the local chemical environment. In fact, it has been a widely accepted strategy of using the monomer to approximately model the polymers in DFT calculations for (electro-)catalysis systems[13-15] and for molecular dynamics simulations.[16] In spite that useful hints have been deduced by our DFT calculations, we should admit that such kind of simplification somewhat ignores the stereo-effect of the –[CHR-CH_2]-group on stabilizing the single-atom catalysts. However, it is worthy of pointing out that the DFT simulations would become infeasible quickly by increasing the system size. In order to study the stereo-effects of the PVP ligands, some kinds of state-of-the-art lower-scaling techniques with a massive-parallel efficiency must be employed.[12, 17] We leave a systematic study on this issue in the near future.

The binding energy ΔE(Pt-PVP@TNR@GC) of platinum on graphene (Pt@G) with *n* PVP on graphene (PVP@G) was calculated as:

$$\Delta E(\text{Pt-}n\text{PVP@G}) = E_t(\text{Pt-}n\text{PVP@G}) - E_t(\text{Pt@G}) - nE_t(\text{PVP@G}) + nE_t(\text{G}) \quad \text{(S1)}$$

where E_t(Pt-*n*PVP@G), E_t(Pt@G), E_t(PVP@G) and E_t(G) are the total energies of the Pt-*n*PVP@G, Pt@G, PVP@G and graphene.

The total hydrogen evolution reaction can be written as $H^+ + e^- \rightarrow 1/2H_2$. At equilibrium, the free energy per H atom (the chemical potential) of the initial and final states of reaction are the same. The free energies of the intermediates were obtained by

$$\Delta G(\text{A-H}) = G_t(\text{A-H}) - G_t(\text{A}) - 0.5G_t(\text{H}_2) \quad \text{(S2)}$$

where G_t(A-H), G_t(A) and G_t(H_2) are the total free energies of the H* adsorption active site,

the active sites, and hydrogen in the gas phase, respectively. Here, we assumed that in addition to the total electronic energies, the translation and rotation contributions of the gas phase H_2 are significant while the other parts can be ignored. And only the vibration contributions of gas phase H_2 and the adsorbate (H*) are considered. Assuming the gas phase H_2 to be an ideal gas, the partition functions of translation Q^{trans} and rotation Q^{rot} were calculated as:[18]

$$Q^{trans} = \left(\frac{2\pi m\, k_B T}{h^2}\right)^{\frac{3}{2}} V \tag{S3}$$

$$Q^{rot} = \frac{1}{\sigma}\frac{k_B T}{hB^{rot}} \text{ (linear)} \tag{S4}$$

where P (1 atm) and m are the pressure and molecular mass, respectively, while k_B is the Boltzmann constant and T (298.15 K) is the absolute temperature. $V = \frac{k_B T}{P_A}$ is the volume of the system, σ is the symmetry factor, B^{rot} are rotational constants, h is the Plank's constant. And the partition functions of vibration Q^{vib} were calculated[11]

$$Q^{vib} = \prod_K \frac{e^{-h\nu_K/2k_B T}}{1-e^{-h\nu_K/k_B T}} \tag{S5}$$

Where ν_K is the frequency of Kth vibration mode. For gas phase hydrogen, there are only one vibration mode and it is calculated as 4305 cm^{-1}. For H* on Pt(111), the frequency of three vibration modes are 1121 cm^{-1}, 555 cm^{-1}, 547 cm^{-1}. For H* on Pt/G, the frequency of three vibration modes are 2099 cm^{-1}, 471 cm^{-1}, 81 cm^{-1}. For H* on Pt-2PVP/G, the frequency of three vibration modes are 2366 cm^{-1}, 730 cm^{-1}, 347 cm^{-1}.

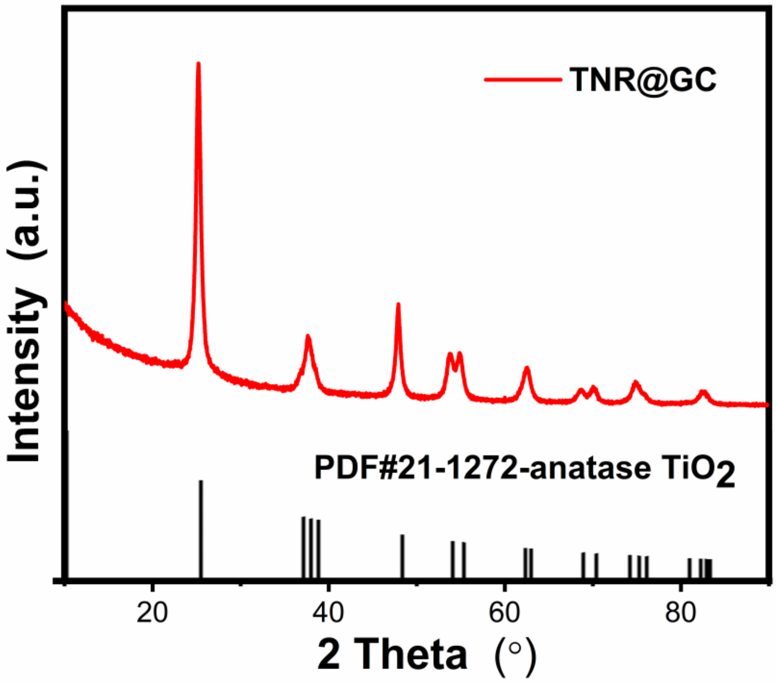

Figure S1 Powder X-ray diffraction (XRD) patterns of graphite carbon encapsulated TiO_2 nanotube (TNR@GC), showing a typical anatase TiO_2 phase.

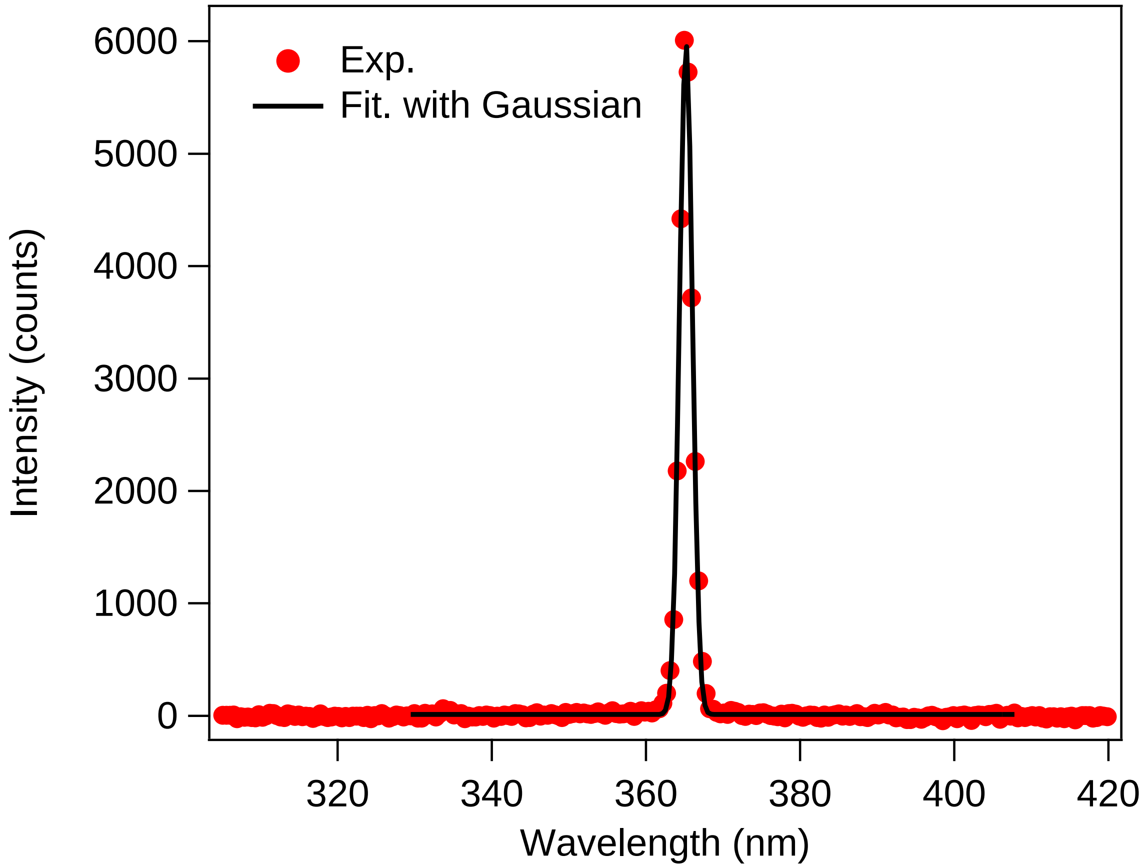

Figure S2 Spectrum of the UV lamp that is used in reduction of Pt^{4+} from the solution. Doted trace is the experimental observation and the solid line is the fit to a Gaussian lineshape function. A center frequency of 365nm was determined with a full width of the half maximum (FWHM) of 1.95 nm.

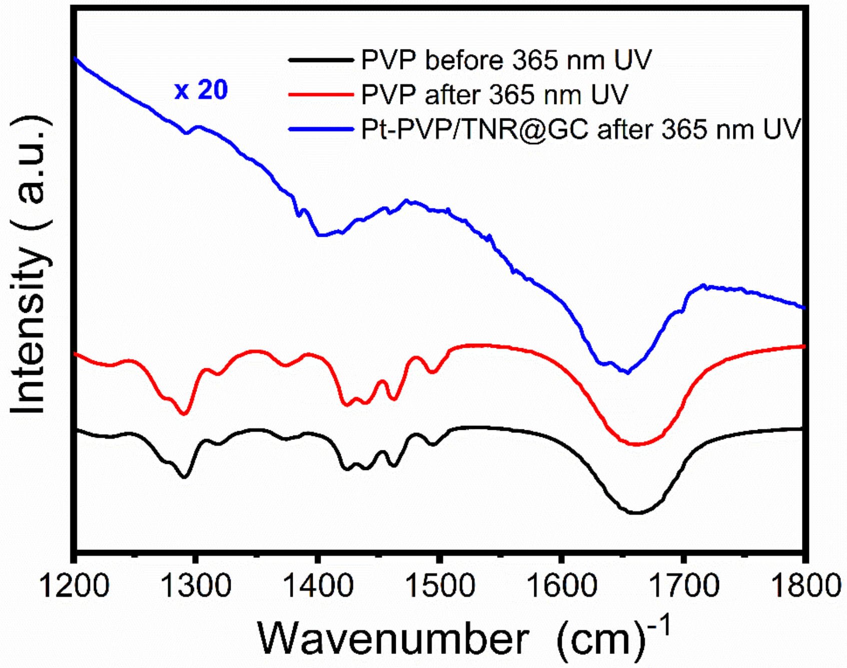

Figure S3. FTIR spectra of pure PVP before (black curve) and after UV (red curve) and Pt-PVP/TNR@GC after UV (blue curve) irradiation. The presence of PVP on the catalysts after UV irradiation can be confirmed by the appearance of the C=O modes in the FTIR spectrum even though the signal to noise ratio is not as great as those of the pure PVP and the spectrum is distorted by the glassy carbon.

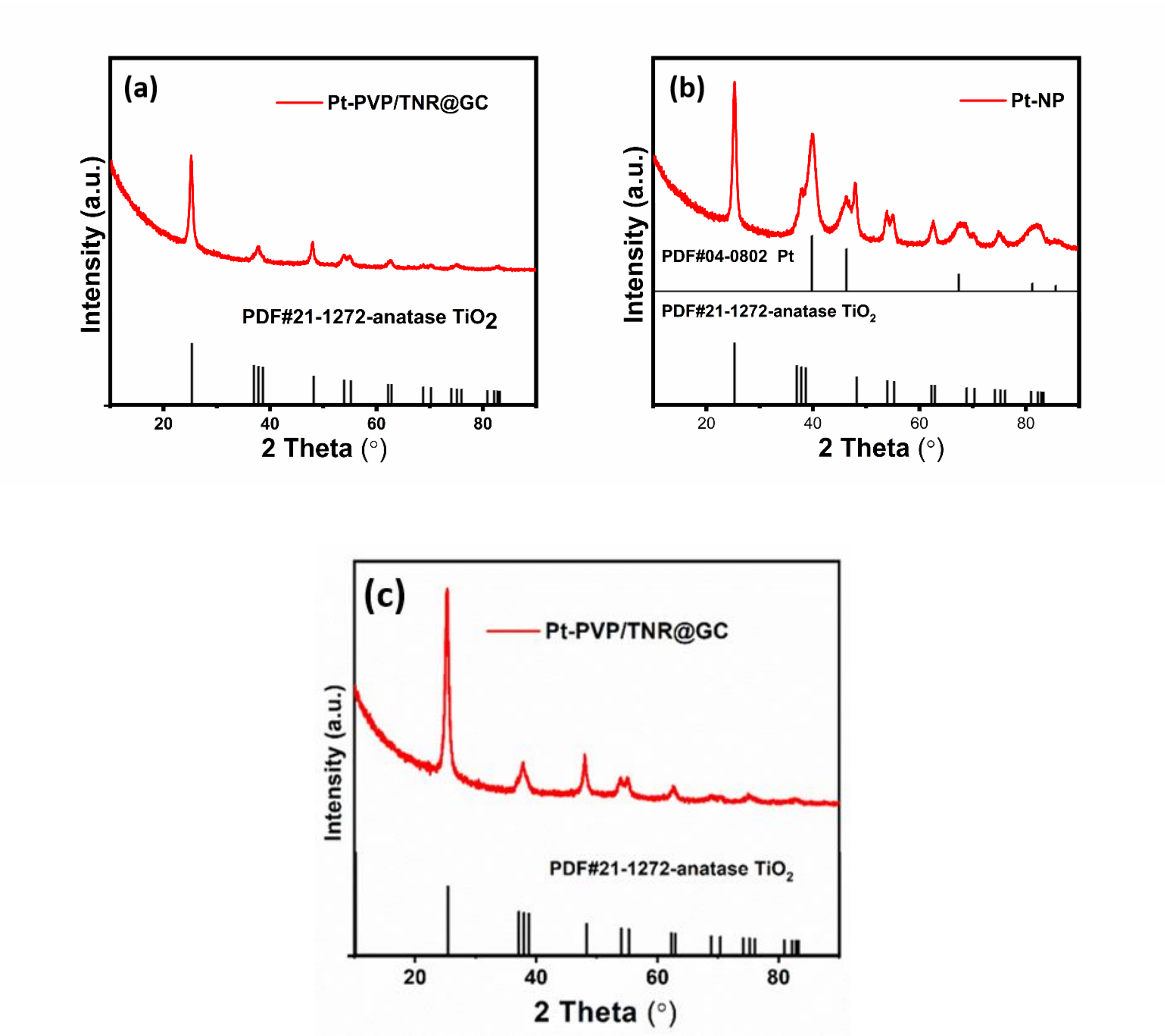

Figure S4 Powder X-ray diffraction (XRD) of patterns of (a) PVP coordinated Pt single atom catalyst on graphite carbon encapsulated TiO_2 nanotube (Pt-PVP/TNR@GC), (b) Pt nanoparticles (Pt-NP) on TNR@GC, and (c) Pt-PVP/TNR@GC after long-term electrochemical durability testing.

The observed reflections of (a) and (c) shows only anatase TiO_2 phase without any Pt nanoparticles characteristic peaks. In comparison to (a) and (c), (b) shows the typical anatase TiO_2 and the (110) crystal type Pt, with sharp peaks located at 39.7 °, 46.2 °, 67.4 ° and 81.3 °, respectively.

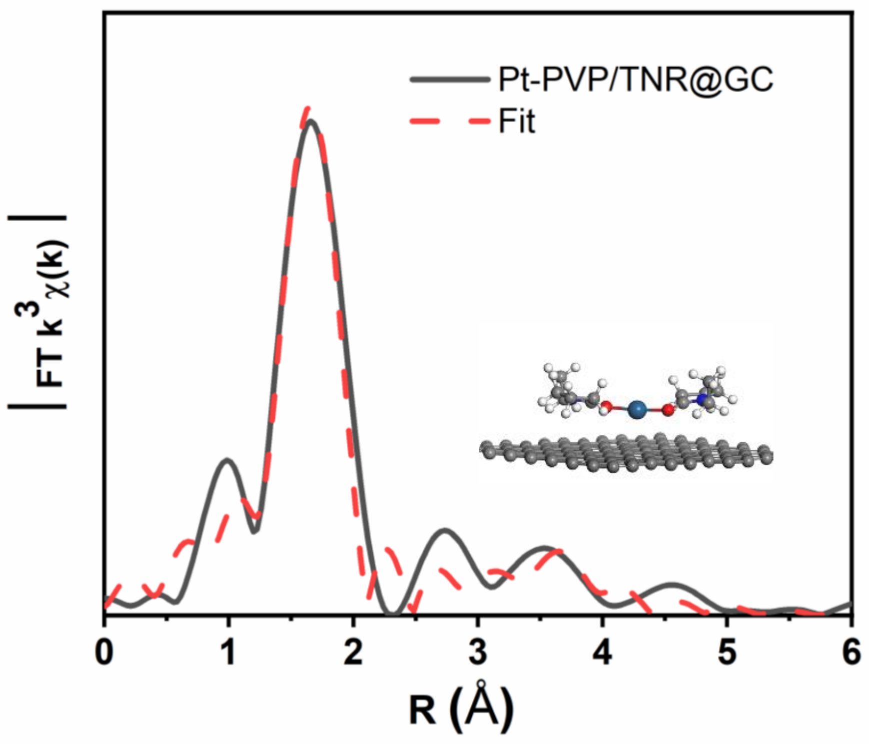

Figure S5 Corresponding EXAFS fitting curve for Pt-PVP/TNR@GC. The DFT calculation results were used to fit the experimental EXAFS results. In Pt-PVP/TNR@GC structure, there are two groups coordinated to Pt atom: two monomers of PVP with Pt-O distances 2.06 Å.

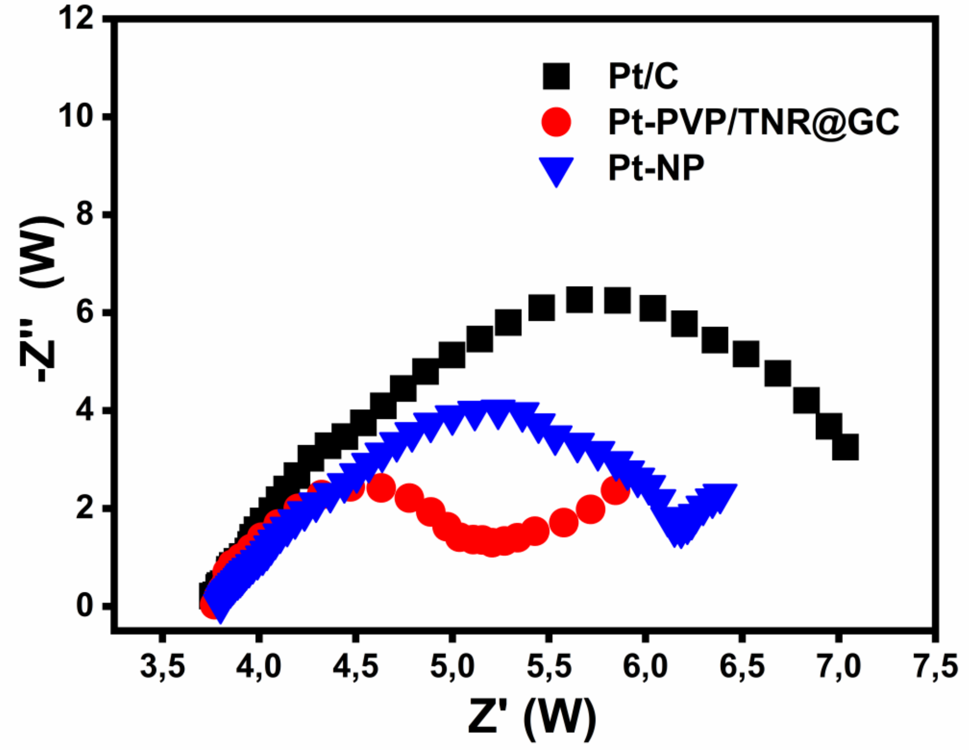

Figure S6 EIS Nyquist plots for various catalysts in 0.5M H_2SO_4. All the potentials have been iR corrected according to the electrochemical impedance spectroscopy (EIS) curves.

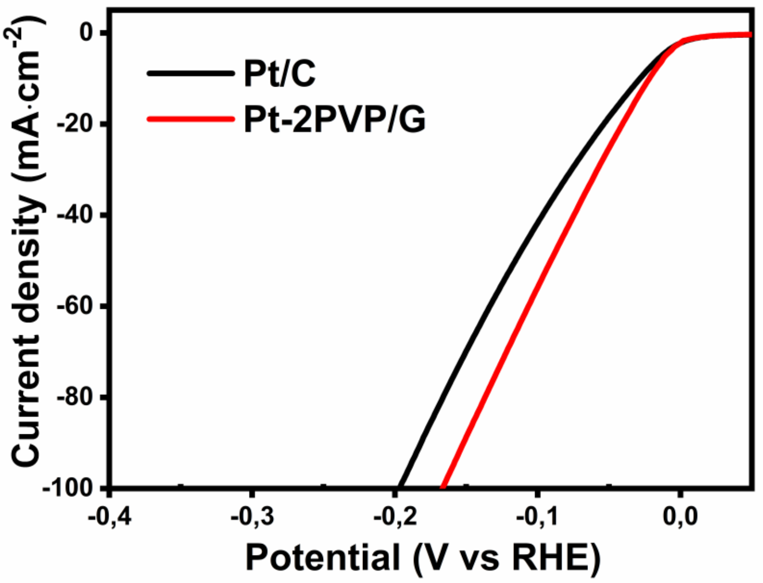

Figure S7 The HER linear sweep voltammetry (LSV) curves of the Pt-PVP/TNR@GC and commercial Pt/C in 1 M KOH media, the Pt-PVP/TNR@GC also exhibits a higher HER activity than Pt/C.

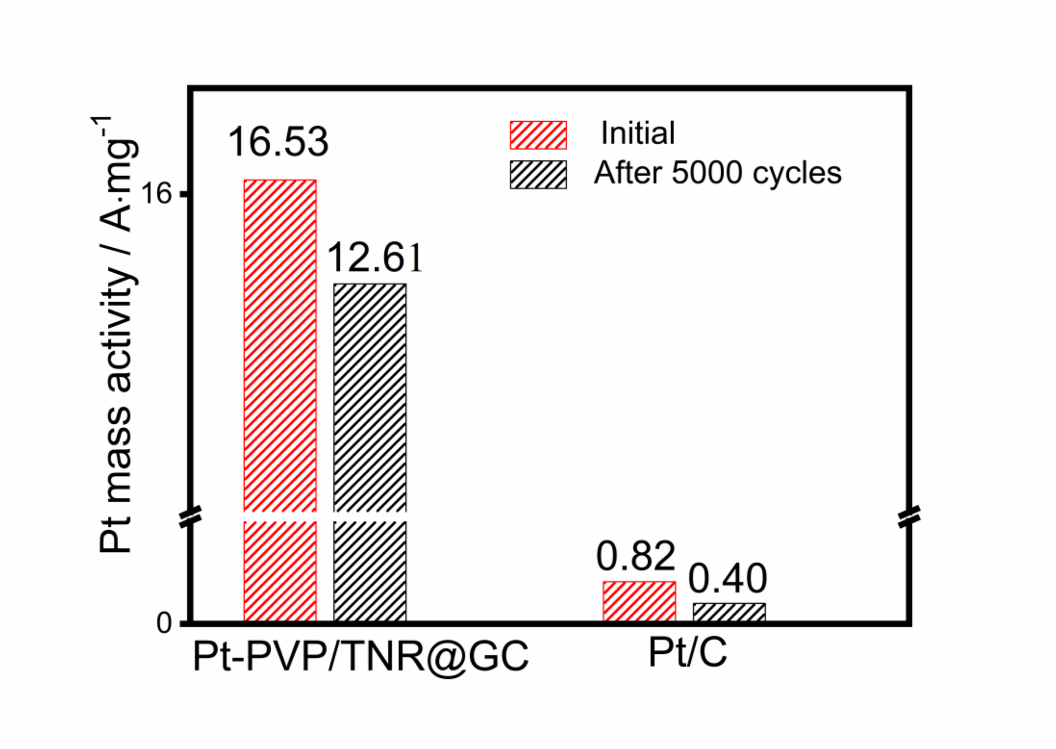

Figure S8 Mass activities at -0.05 V of the Pt-PVP/TNR@GC and commercial Pt/C for the HER before and after 5,000 cycles. The as-prepared Pt-PVP/TNR@GC unfolds remarkable kinetic activities (20 times higher in current density than the best commercially available Pt/C) with excellent stability (76.3% of its initial activity after 5000 cycles) for HER

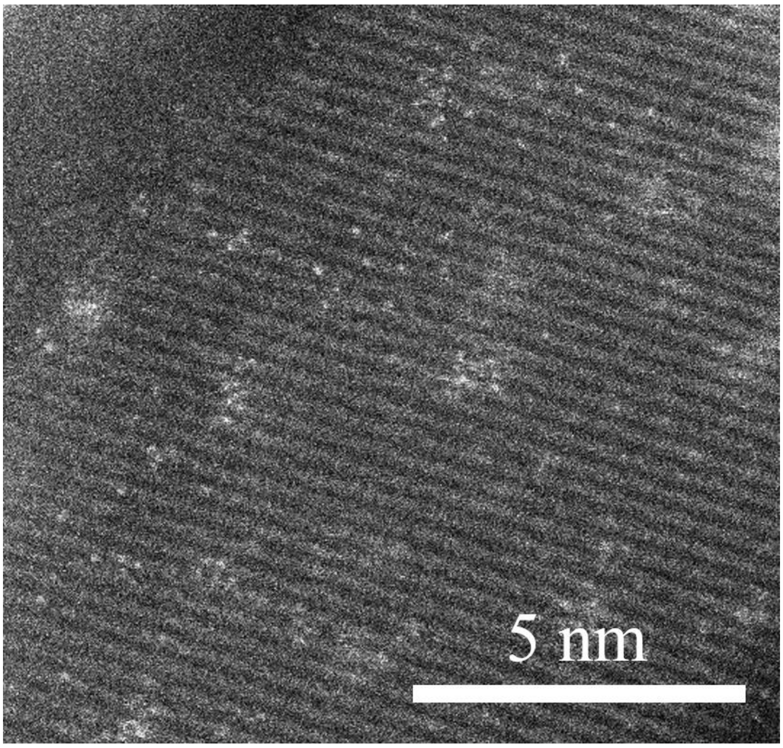

Figure S9 After long-term durability testing HAADF-STEM image of Pt-PVP/TNR@GC. The atomically isolated dispersed Pt atoms remain, and no Pt nanoparticles are observed.

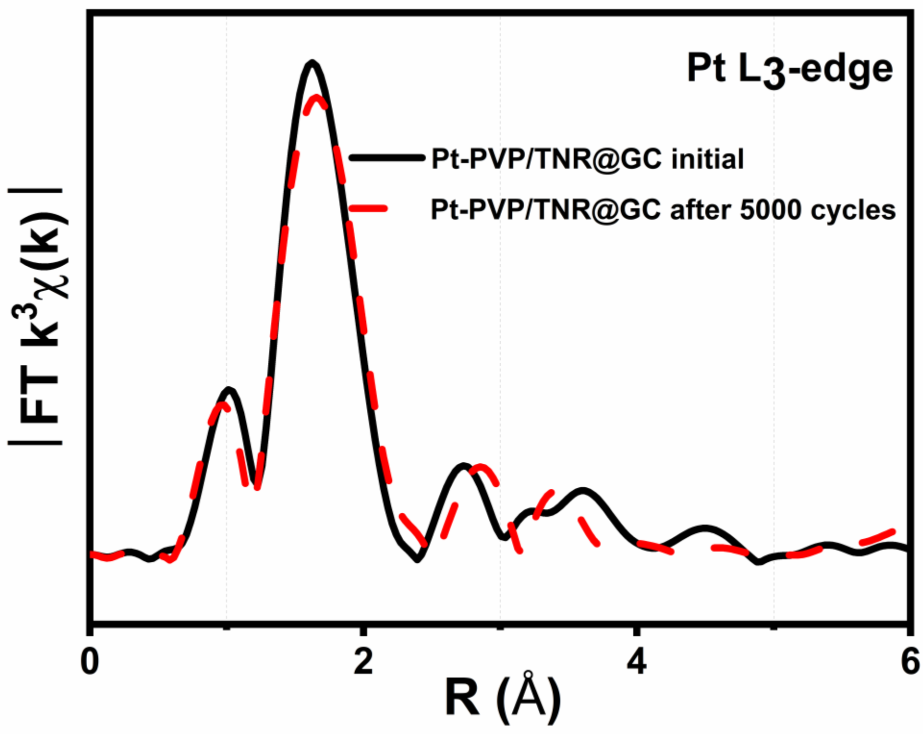

Figure S10 Before and after long-term durability testing EXAFS of Pt-PVP/TNR@GC, exhibiting only one prominent peak around 1.65 Å, without Pt-Pt bond peaks.

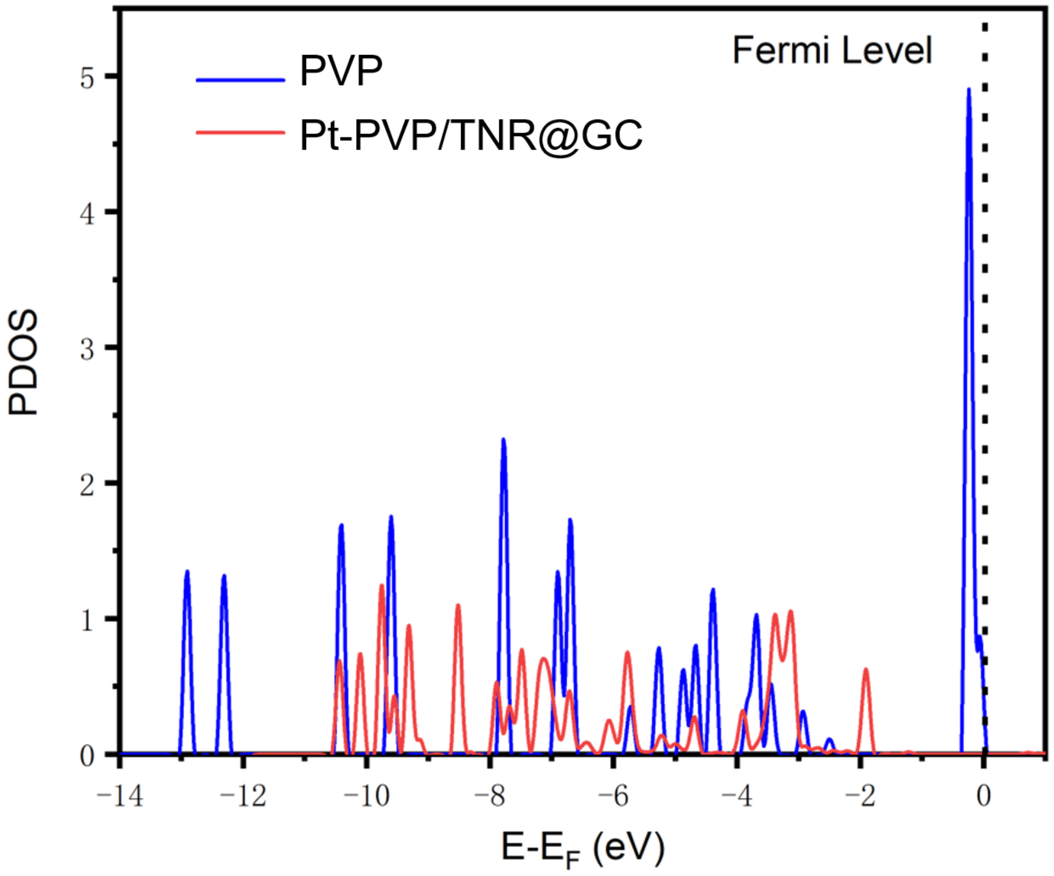

Figure S11. The partial density of state (PDOS) of N in gas phase PVP and in Pt-PVP@GC. The lone pair electrons peak of N atoms in Pt-PVP@GC disappeared, which can be attributed to N1s→π* transitions.

Table S1. N atom concentration on the surface of Pt-PVP/TNR@GC samples.

Sample No	PVP amount	TNR@GC	Pt precursor	N atom concentration
1	15 mg	20 mg	75uL	2.90 %
2	30 mg	20 mg	75uL	3.64 %
3	60 mg	20mg	75uL	3.36 %

Table S2. EXAFS parameters of samples Pt-PVP/TNR@GC.

Sample	Scattering pair	CN	R(Å)	σ^2	ΔE_0(eV)	Delta R
Pt-PVP/TNR@GC.	Pt-O	2	2.06	0.00627	14.707	0.04185
Pt-PVP/TNR@GC.	Pt-C_{pvp}	2	3.69	0.02188	14.707	0.42834
Pt-PVP/TNR@GC.	Pt-N	2	4.20	0.00411	14.707	0.00110

Table S3 DFT results of bond length changes of Pt-PVP/TNR@GC in comparison with pure PVP

	Distance of C=O	Distance of C-N
PVP	1.23 Å	1.27 Å
Pt-PVP/TNR@GC	1.27 Å	1.34 Å

The N-C bond the N-C bond length shorten from 1.37 Å to 1.34 Å, and the C=O bond length expanded from 1.23 Å to 1.27 Å, as the PVP is coordinated with Pt atom

Reference

[1] G. Kresse, J. Furthmüller, *Comput. Mater. Sci.* **1996**, *6*, 15.

[2] G. Kresse, J. Furthmüller, *Phys. Rev. B* **1996**, *54*, 11169.

[3] G. Kresse, J. Hafner, *Phys. Rev. B* **1993**, *48*, 13115.

[4] P. E. Blöchl, *Phys. Rev. B* **1994**, *50*, 17953.

[5] H. J. Monkhorst, J. D. Pack, *Phys. Rev. B* **1976**, *13*, 5188.

[6] J. P. Perdew, K. Burke, M. Ernzerhof, *Phys. Rev. Lett.* **1996**, *77*, 3865.

[7] S. Grimme, J. Antony, S. Ehrlich, H. Krieg, *J. Chem. Phys.* **2010**, *132*, 154104.

[8] S. Grimme, S. Ehrlich, L. Goerigk, *J. Comput. Chem.* **2011**, *32*, 1456.

[9] G. Henkelman, A. Arnaldsson, H. Jónsson, *Comput. Mater. Sci.* **2006**, *36*, 354.

[10] E. Sanville, S. D. Kenny, R. Smith, G. Henkelman, *J. Comput. Chem.* **2007**, *28*, 899.

[11] W. Kohn, *Phys. Rev. Lett.* **1996**, *76*, 3168.

[12] I. Y. Zhang, X. Xu, Y. Jung, W. A. Goddard, *Proc. Natl. Acad. Sci.* **2011**, *108*, 19896.

[13] S. Ye, F. Luo, Q. Zhang, P. Zhang, T. Xu, Q. Wang, D. He, L. Guo, Y. Zhang, C. He, X. Ouyang, M. Gu, J. Liu, X. Sun, *Energy Environ. Sci.* **2019**, *12*, 1000.

[14] A. M. Abdelghany, M. S. Mekhail, E. M. Abdelrazek, M. M. Aboud, *J. Alloys Compd.* **2015**, *646*, 326.

[15] J.-Y. Ye, G. A. Attard, A. Brew, Z.-Y. Zhou, S.-G. Sun, D. J. Morgan, D. J. Willock, *J. Phys. Chem. C.* **2016**, *120*, 7532.

[16] A. Gupta, B. Boekfa, H. Sakurai, M. Ehara, U. D. Priyakumar, *J. Phys. Chem. C.* **2016**, *120*, 17454.

[17] W. Guo, A. Wu, I. Y. Zhang, X. Xu, *J. Comput. Chem.* **2012**, *33*, 2142.

[18] I. Chorkendorff, J. W. Niemantsverdriet, *Concepts of modern catalysis and kinetics*, John Wiley & Sons, **2017**.
